# IECA-YOLOv7: A Lightweight Model with Enhanced Attention and Loss for Aerial Wildlife Detection

**DOI:** 10.3390/ani15182743

**Published:** 2025-09-19

**Authors:** Wenyue Ke, Tengfei Liu, Xiaohui Cui

**Affiliations:** 1 College of Information Science and Technology, Beijing Forestry University, Beijing 100083, China; 19971718030@163.com (W.K.); l980336239@bjfu.edu.cn (T.L.); 2 Engineering Research Center for Forestry-Oriented Intelligent Information Processing of National Forestry and Grassland Administration, Beijing 100083, China; 3 Hebei Key Laboratory of Smart National Park, Beijing Forestry University, Beijing 100083, China

**Keywords:** grassland wildlife, aerial imagery, lightweight model, attention mechanism, small target detection

## Abstract

Grassland ecosystems are vital for global biodiversity, yet traditional wildlife monitoring methods are often labor-intensive and costly. Although drone-based aerial surveys provide a scalable alternative, they face significant challenges such as detecting extremely small targets, handling complex backgrounds, and operating under strict computational constraints. To address these issues, this study proposes IECA-YOLOv7, a lightweight detection model that incorporates three key innovations: an Improved Efficient Channel Attention (IECA) module for enhanced feature representation, a content-aware CARAFE upsampling operator for improved detail recovery, and a Normalized Wasserstein Distance (NWD) loss function for robust small-target localization. Evaluated on a dedicated grassland wildlife dataset (GWAID), the model achieves a mAP@0.5 of 86.6% and a mAP@0.5:0.95 of 47.2%, outperforming the baseline YOLOv7-tiny by 2.9% in Precision and 1.8% in Recall. Furthermore, it surpasses non-YOLO architectures such as RetinaNet, EfficientDet-D0, and DETR by significant margins, demonstrating superior performance in small-object detection under complex conditions. Cross-dataset validation on VisDrone, CARPK, and DOTA demonstrates a strong generalization capability. With a model size under 5 MB, IECA-YOLOv7 effectively balances accuracy and efficiency, offering a practical solution for real-time wildlife monitoring via drones under challenging environmental constraints such as variable lighting, occlusion, and limited computational resources, thereby supporting broader conservation efforts.

## Simple Summary

Traditional wildlife monitoring in grasslands is inefficient and costly, especially when using drones to detect animals from the air. Challenges include animals appearing very small in images, blending into complex backgrounds like grass and shadows, and the need for lightweight computer models that can run on drones. To solve this, we developed a new lightweight detection system called IECA-YOLOv7. It uses an improved attention module to help the model focus on key animal features, a smarter upscaling method to preserve details in small objects, and a specialized loss function to better locate tiny targets. Tested on aerial images of grassland wildlife (such as antelopes and zebras), our model achieved 86.6% detection accuracy, outperforming existing lightweight methods. This system provides an efficient, low-cost tool for ecologists to monitor wildlife populations and support conservation efforts.

## 1. Introduction

Grassland ecosystems, spanning over 40% of the Earth’s terrestrial surface, serve as critical reservoirs of biodiversity and carbon sequestration, underpinning global ecological stability and climate resilience [1]. Yet monitoring wildlife within these vast, often remote regions remains notoriously inefficient. Traditional approaches—such as manual field surveys and camera traps [2]—are labor-intensive, costly, and spatially limited, hindering large-scale conservation efforts. The emergence of uncrewed aerial vehicles (UAVs) offers a transformative solution for aerial surveillance [3,4], enabling rapid, non-invasive data collection across expansive grasslands. In recent years, UAV-based wildlife monitoring has gained significant traction, with advances in deep learning enhancing automated detection capabilities. For example, recent studies by Smith et al. [5] utilized multi-spectral UAV imagery and a transformer-based detector to achieve real-time animal identification in savanna environments, demonstrating high accuracy under varying lighting conditions. Similarly, Chen et al. [6] proposed an attention-guided network for detecting small and occluded wildlife in grassland UAV videos, significantly reducing false positives through spatial-temporal feature fusion. Furthermore, Osco et al. [7] provided a broad review of deep learning applications in UAV remote sensing, covering various tasks beyond wildlife monitoring, such as vegetation classification, regression, and segmentation, while also analyzing trends across a large body of literature. Despite these advancements, many existing methods still face challenges in balancing accuracy and computational efficiency for real-time deployment on resource-constrained platforms.

The integration of remote sensing and ecological informatics has further expanded the potential of UAVs in conservation biology [7,8]. Recent advances in deep learning have led to the development of sophisticated models for processing aerial imagery. For example, Faster R-CNN with Feature Pyramid Networks (FPN) has been widely adopted for its strong performance in multi-scale object detection [9], while Vision Transformers (ViTs) and Swin Transformers have shown remarkable success in capturing long-range dependencies in high-resolution images [10,11]. These models have been applied in various wildlife monitoring contexts, such as species identification [12], population counting [13], and behavior analysis [14]. However, despite their successes, these state-of-the-art methods face significant limitations when applied to UAV-based wildlife detection in grassland environments.

Automated detection of wildlife in UAV imagery confronts three persistent challenges: (a) extreme small-target scales (typically 0.03–0.15% of image pixels at 80–150 m altitude), which exacerbate feature loss during convolutional downsampling [15,16]; (b) complex background interference (e.g., dense vegetation, shadows, and occlusions) causing false-positive rates exceeding 55%; and (c) stringent computational constraints of UAV platforms, demanding lightweight models (<5 MB) without sacrificing accuracy. While some recent studies have begun to address these issues—for example, Lyu et al. [17] employed thermal imagery and an enhanced Faster R-CNN for deer detection, improving accuracy under certain conditions—their models remain computationally heavy and are not optimized for real-time drone deployment. Similarly, Wang et al. [18] combined deep learning with species distribution models for giant panda detection, yet their approach was tailored to specific species and environments, limiting generalizability.

Existing deep learning approaches, including transformer-based architectures and two-stage detectors like Faster R-CNN, often struggle with the unique challenges posed by aerial wildlife monitoring. While these models excel in general object detection tasks, they are typically designed for larger targets and fail to maintain performance when applied to extremely small objects (often <20 × 20 pixels) characteristic of UAV imagery [19,20]. Furthermore, their substantial computational requirements (often hundreds of megabytes to several gigabytes) make them unsuitable for real-time deployment on resource-constrained UAV platforms [21,22]. The Swin Transformer, despite its powerful representation learning capabilities, suffers from high computational complexity and memory usage, limiting its practicality for onboard processing [23]. Similarly, Vision Transformers applied to drone imagery require extensive computational resources and lack optimization for the specific challenges of wildlife detection in complex grassland backgrounds [24].

While recent advances in real-time object detection have yielded compact architectures like YOLOv7-tiny (6.2 M parameters), its direct application to aerial wildlife monitoring reveals critical shortcomings. First, shallow layers lack the capacity to capture the discriminative features of diminutive targets against cluttered backgrounds. Second, nearest-neighbor upsampling in the neck network discards contextual information vital for small-object recovery. Third, the IoU (Intersection over Union) loss function exhibits a poor robustness to micro-scale localization errors—a well-documented flaw in aerial detection [25,26]. Though attention mechanisms (e.g., SE, CBAM) as well as feature fusion strategies have been proposed to enhance small-object sensitivity [27,28,29], these approaches struggle in grasslands where target-background homogeneity and sparse activations dominate.

Controversially, some studies argue that lightweight models inherently trade accuracy for efficiency [30,31], while others posit that architectural innovations can bridge this gap [32,33]. This divergence underscores the need for targeted solutions that reconcile computational efficiency with ecological specificity.

This study aims to address these critical gaps by developing a specialized lightweight detection framework that balances computational efficiency with high accuracy for small-target wildlife detection in aerial grasslands. Our research is motivated by three specific scientific problems: (1) how to enhance feature representation for extremely small targets (0.03–0.15% of image pixels) while maintaining computational efficiency suitable for UAV deployment; (2) how to improve localization accuracy for overlapping and occluded wildlife in complex grassland backgrounds; and (3) how to develop a unified framework that integrates these advancements while remaining under 5 MB for practical drone implementation.

To solve the gaps, we introduce IECA-YOLOv7—a new detection framework optimized for aerial wildlife scenarios. Our contributions are threefold: (a) IECA-ELAN Module: An Improved Efficient Channel Attention (IECA) mechanism incorporating parallel global max pooling (GMP) as well as average pooling (GAP) branches to amplify multi-scale feature recalibration. Integrated with ELAN networks, it dynamically prioritizes discriminative small-target features while suppressing background noise; (b) CARAFE Upsampling: Replacing naive interpolation with content-aware feature recombination, this operator adaptively generates kernels to preserve critical spatial context during resolution recovery, and (c) NWD Loss Function: By modeling bounding boxes as Gaussian distributions (GD) as well as leveraging NWD, we mitigate IoU’s sensitivity to minute localization errors in tiny targets [26].

The proposed IECA-YOLOv7 framework specifically addresses the limitations of existing approaches in several key aspects, and our contributions are threefold: (a) Compared to transformer-based methods, our model maintains high accuracy for small targets while reducing computational requirements by over 90%; (b) unlike generic attention mechanisms (SE, CBAM), our IECA module specifically enhances feature discrimination in low-contrast grassland environments; and (c) compared to standard YOLO variants, our integration of CARAFE upsampling and NWD loss significantly improves localization accuracy for overlapping and small targets. These innovations collectively enable real-time wildlife monitoring without sacrificing detection performance.

Comprehensive validation on a dedicated grassland wildlife dataset (GWAID) and public benchmarks (VisDrone, CARPK, and DOTA) confirms our approach’s efficacy. IECA-YOLOv7 achieves state-of-the-art accuracy (86.6% mAP@0.5) while retaining deployability on edge devices. This work not only advances UAV-based conservation but also resolves key controversies in lightweight small-object detection.

The core objectives of this study are as follows: (1) to develop and validate an improved channel attention mechanism specifically optimized for small wildlife target detection in aerial imagery; (2) to integrate content-aware upsampling and distribution-based loss functions to enhance localization accuracy; (3) to create a lightweight framework (<5 MB) suitable for real-time UAV deployment; and (4) to demonstrate a superior performance compared to existing state-of-the-art methods across multiple datasets and application scenarios.

## 2. Materials and Methods

The selection of methodologies in this study was driven by the specific challenges of aerial wildlife detection: extremely small-target sizes, complex grassland backgrounds, and the need for lightweight, real-time capable models. We opted for an integrated approach that combines attention mechanisms, advanced upsampling, and distribution-based loss functions to address these issues systematically. Each component was chosen based on its proven efficacy in related computer vision tasks and its adaptability to the ecological context of UAV-based monitoring. The following subsections detail not only the implementation of these methods but also the rationale behind their selection, emphasizing their suitability for small-target detection in cluttered aerial imagery.

**Figure 1:**
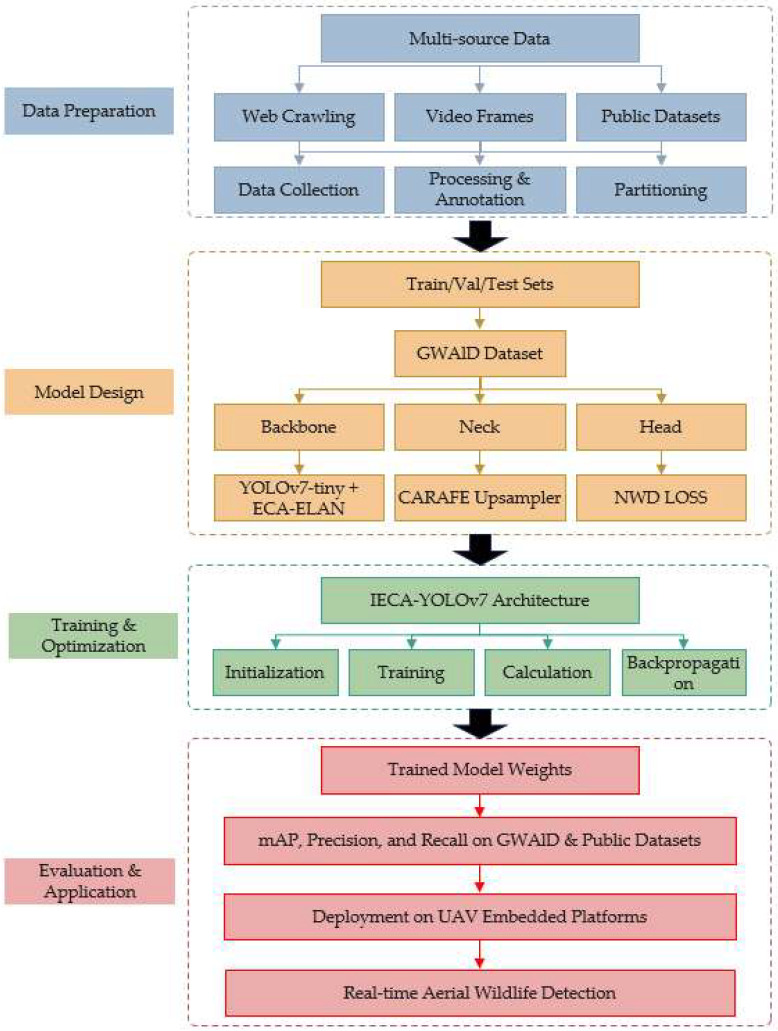
Overall workflow of the IECA-YOLOv7 system for aerial wildlife detection.

To provide a clear overview of the methodological framework of this study, Figure 1 illustrates the general workflow of the proposed IECA-YOLOv7 system, from data collection to model deployment. The process encompasses four main stages: (1) data acquisition and annotation, (2) model architecture design, (3) training and optimization, and (4) evaluation and application. Each stage is composed of multiple detailed steps, which are elaborated in the subsequent subsections.

As shown in Figure 1, the workflow begins with the construction of a specialized grassland wildlife dataset (GWAID), which involves multi-source data collection, rigorous annotation, and strategic partitioning. The core model improvements—IECA mechanism, CARAFE upsampling, and NWD loss—are integrated into the YOLOv7-tiny backbone. The model is then trained and validated under carefully configured experimental settings, with comprehensive evaluations conducted on both self-built and public datasets. The following subsections detail each component of this pipeline.

### 2.1. Construction of Aerial Grassland Wildlife Image Dataset

#### 2.1.1. Study Area Context

To provide a clear ecological and operational context for the dataset and subsequent model development, it is essential to define the study environment. This research is grounded in the context of monitoring wildlife in typical grassland and savanna ecosystems, such as those found in the Serengeti National Park (Tanzania), Maasai Mara National Reserve (Kenya), and the Eastern Mongolian steppes. Geographically, these regions are characterized by vast, open plains, with altitudes ranging from 1000 to 2000 m, interspersed with acacia woodlands and rocky kopjes in East Africa, and gently rolling hills in the Mongolian steppes. The climate is predominantly tropical savanna in East Africa, with distinct dry (June–October) and wet (November–May) seasons, and cold semi-arid in Mongolia, with precipitation patterns greatly influencing vegetation phenology and animal distribution.

The biophysical context involves monitoring keystone herbivore species (e.g., wildebeest, zebra, and Thomson’s gazelle) and mega-fauna (e.g., African elephant, giraffe) that are crucial for maintaining ecosystem health through grazing and as a foundation for tourism. The core detection challenges are directly tied to this environment: animals often exhibit cryptic coloration that blends with dry-season grasses, are partially obscured by dense vegetation or shrubs, and are cast in dappled shadows under scattered trees. Targets are rarely completely occluded but are frequently visually ambiguous.

The socio-economic background is driven by the need for cost-effective, large-scale population surveys to inform conservation policies and anti-poaching efforts in protected areas that are often under-resourced. These regions are primarily designated as national parks or community conservancies. Economic activities in surrounding areas include pastoralism and wildlife tourism, which can create human–wildlife conflict. The development of automated monitoring tools aims to replace traditional aerial surveys that are both labor-intensive, costly, and logistically challenging, thereby providing more frequent and accurate data for biodiversity assessment and management decisions.

#### 2.1.2. Data Collection and Annotation Framework

To address the lack of specialized datasets for aerial grassland wildlife detection, the research proposes a comprehensive framework for constructing an aerial wildlife dataset, encompassing five key modules: data collection, data processing, data annotation, data partitioning, and dataset assembly. The framework employs standardized aerial imaging protocols for image acquisition, ensures data quality through professional processing, establishes multi-level annotation specifications for accuracy, utilizes stratified sampling strategies for data division, and implements a closed-loop optimization system. Based on this framework, we constructed the GWAID (Grassland Wildlife Aerial Images from Drone) dataset, the first standardized dataset specifically designed for aerial grassland wildlife detection. The dataset comprises a total of 10,081 high-quality aerial images, meticulously collected and annotated, featuring five representative species (antelope, elephant, sheep, giraffe, and zebra).

Although the precise geolocation and capture timestamp for every image were not systematically recorded due to the multi-source nature of the data collection, the dataset was explicitly constructed to encapsulate a wide qualitative diversity. The imagery was gathered from various nature reserves across East Africa (e.g., Serengeti, Maasai Mara) and Central Asia (e.g., Mongolian steppes) between 2018 and 2023. It encompasses a wide range of seasonal (dry and wet seasons) and lighting conditions (dawn, daylight, dusk, and overcast), as evidenced by the varying vegetation lushness, ground color, and shadow lengths. This deliberate heterogeneity ensures that the GWAID possesses the ecological validity necessary for training a robust detection model. The original sensor resolutions of the collected imagery vary, including common standards such as 1920 × 1080, 3840 × 2160, and 4000 × 3000 pixels, prior to our standardized preprocessing resizing to 640 × 640 pixels for model training.

This approach was preferred over using generic aerial datasets (e.g., COCO) because it captures the specific challenges of grassland environments—such as partial occlusions, shadowing, and low contrast—which are critical for training robust detectors. The five-species focus was chosen to represent a range of sizes and shapes commonly encountered in savanna and grassland ecosystems, thereby enhancing the model’s practical applicability.

### 2.2. Collection and Processing of Aerial Grassland Wildlife Images

This study employed a multi-source heterogeneous data fusion strategy to build a diverse library of aerial wildlife images covering extensive scenarios. The imagery was curated to reflect real-world operational conditions, where monitoring missions are conducted over protected areas with diverse topography—from flat grasslands to moderately complex, rugged landscapes. The imagery was specifically curated to include a wide spectrum of environmental conditions critical for robust model generalization. This includes variations in the following: (a) Illumination: Bright sunlight, partial cloud cover, overcast conditions, and low-light scenarios during dawn/dusk; (b) Seasonality: Landscapes from both dry and rainy seasons, resulting in changes in vegetation density and ground color; (c) Occlusion: Targets partially obscured by grasses, shrubs, shadows, or terrain features; and (d) Sensor Characteristics: Images were sourced from various UAV-mounted cameras (e.g., DJI Zenmuse P1, X7, etc.; manufacturer: DJI, Shenzhen, China), leading to a diversity of original resolutions ranging from 2 to 24 megapixels before uniform resizing for model input.

The data collection process integrated three technical pathways: web crawling, video frame extraction, and migration from public datasets, forming an end-to-end workflow from raw data acquisition to standardized processing. The final dataset includes diverse samples captured under different lighting conditions, flight altitudes, and geographical distributions, effectively reflecting the complexity of real-world grassland ecological monitoring scenarios.

This multi-source strategy was prioritized over synthetic data generation to ensure that the training data reflects real-world variability in lighting, altitude, and occlusion. This approach is better suited for generalization than simulated data, which often fails to capture the noise and complexity of actual field conditions.

#### 2.2.1. Web Scraping and Image Filtering

A keyword list (e.g., “aerial wildlife monitoring,” “drone grassland animal tracking”) was used to crawl mainstream search engines (Google, Bing) and wildlife photography websites (e.g., Pixabay). The search was specifically targeted to include scenarios from protected grassland reserves and savannas, ensuring the collected images represented authentic field conditions with natural elements like grass occlusion, dappled shadows under trees, and animals blending into the background. This method gathered a large volume of aerial wildlife-related images from multiple sources. After manual screening and classification, qualified images were categorized by animal species to form a preliminary raw image dataset. Subsequently, duplicate entries and irrelevant content were filtered out to ensure dataset purity and representativeness, resulting in a standardized image dataset.

#### 2.2.2. Video Data Extraction and Image Processing

**Figure 2:**
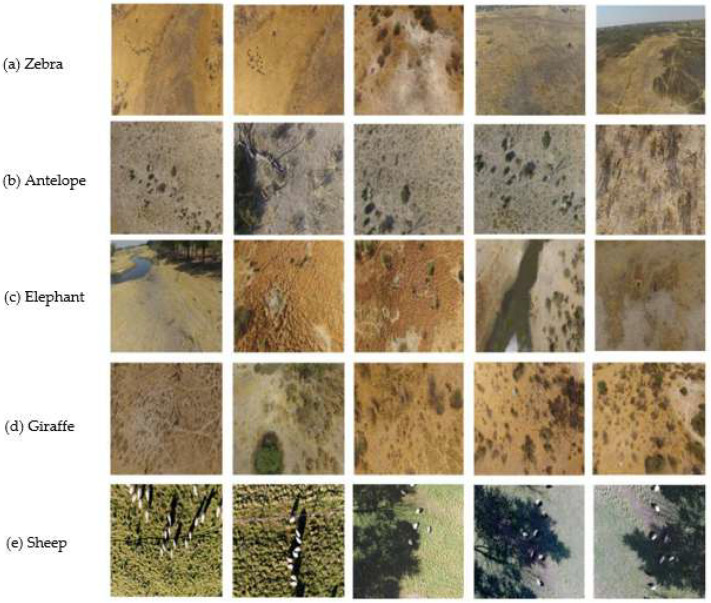
Sample images from the dataset, illustrating the typical environmental conditions, including open plains, light vegetation cover, and partial occlusions.

To handle video data characteristics, an intelligent frame extraction system (comprising Python 3.10 scripts for video reading and interval-based frame sampling) was developed to process aerial wildlife videos from platforms like Bilibili and YouTube. For collected video data, script tools extracted individual frames. Each frame was resized to a uniform resolution (1018 × 572 pixels), balancing image quality and computational efficiency. The processed video frames, combined with web-crawled data, formed a foundational image library covering six typical grassland habitats (e.g., savanna, wetlands, and shrubland). These habitats were selected to represent a gradient of complexity, from open flatlands where targets are clearly visible but very small to shrublands with moderate occlusion, simulating the varying detection challenges encountered in real-world transect surveys. Representative dataset images are displayed in Figure 2.

#### 2.2.3. Acquisition of Public Datasets

In addition to self-collected materials, multiple public data sources were incorporated to enhance data diversity. For instance, aerial wildlife image collections specifically captured by drones were obtained from the Roboflow Universe platform. These materials, shared by global ecological conservation organizations, encompass animal activities across seasons and weather conditions. These public datasets not only include clear animal imagery but also provide detailed metadata (e.g., geographical location, capture time), offering multidimensional references for subsequent analysis and ensuring the model is exposed to a wide range of environmental and operational conditions relevant to conservation practices.

To effectively integrate external data with self-collected materials, data format standardization was performed. Due to variations in annotation formats across platforms (e.g., COCO, PASCAL VOC), an automated conversion tool was developed to unify all annotation files into the YOLO format, ensuring correct model interpretation.

By integrating images from diverse sources, a heterogeneous aerial wildlife image dataset was constructed. This multi-channel collection and processing approach ensured high quality and representativeness, meeting the requirements for model training and evaluation.

Ethical Note on Data Usage: Clarification of dataset usage rights and licensing for both web-scraped and public sources is provided to address possible ethical concerns. For images obtained through web scraping, we strictly adhered to the terms of service of the source websites and prioritized content that was explicitly labeled for reuse under licenses such as Creative Commons. For public datasets (e.g., Roboflow Universe), we ensured that all materials used were publicly available for academic research purposes and complied with their respective citation and licensing requirements. All data in the constructed GWAID dataset is intended solely for non-commercial academic research.

### 2.3. Data Annotation and Partitioning

Data annotation is a core process in building high-quality image datasets, directly impacting subsequent model training accuracy and performance. To meet the requirements of this study’s aerial grassland wildlife image dataset, standardized annotation procedures and data partitioning strategies were implemented to ensure accuracy, representativeness, and diversity, providing a reliable foundation for deep learning model training.

#### 2.3.1. Data Annotation

The professional annotation tool “LabelImg” was used to annotate aerial wildlife images. This tool supports the automatic generation of YOLO-format annotation files, improving annotation efficiency and ensuring format consistency. During annotation, bounding boxes were added to wildlife targets in each image based on spatial location and size information precisely matched to category labels. Detailed annotation specifications defined bounding box coverage, labeling standards, and category divisions to ensure dataset quality.

To guarantee annotation accuracy and consistency, a strict quality control mechanism was established. After every 500 annotated images, results underwent a manual review focusing on accuracy, consistency, and completeness. Images failing to meet standards were re-annotated. After multiple rounds of review and revision, the final annotations ensured high reliability and uniformity.

All generated annotation files were stored in the standard YOLO format, facilitating accurate reading and efficient processing by deep learning models. To enhance dataset versatility, it was also converted to VOC format, enabling richer metadata recording and support for multi-task learning.

#### 2.3.2. Data Partitioning

Scientific dataset partitioning is critical for unbiased algorithm performance evaluation. This study adopted a tripartite split strategy: training (70%), validation (20%), as well as test sets (10%). This ratio aligns with common practices for medium-scale datasets in computer vision (e.g., PASCAL VOC, and ImageNet subsets), ensuring sufficient training samples while reserving independent data for model tuning and final evaluation.

A scripted randomization tool ensured balanced class distribution across training, validation, as well as test sets. For each category, images were randomly assigned according to predefined ratios, guaranteeing comprehensive representation of dataset characteristics across partitions.

This rational partitioning strategy ensured data representativeness, mitigated overfitting risks, and enhanced the model generalization capability in real-world applications. GWAID partitioning results are displayed in Table 1.

This partitioning strategy follows best practices in deep learning for medium-sized datasets and helps prevent overfitting. The choice of the YOLO format was motivated by its efficiency and compatibility with real-time detection frameworks, which aligns with our goal of UAV deployment.

To promote research reproducibility as well as academic exchange, the GWAID dataset has been open sourced on GitHub (Project URL: https://github.com/xiaohuicui/WAID, accessed on 23 January 2025). The repository includes the complete dataset (pre-partitioned), detailed annotation specifications, standardized data loading interfaces, evaluation scripts, and pretrained model weights. Since its release, it has been downloaded over 50 times, supported multiple top-conference papers, and been applied in several wildlife monitoring projects.

### 2.4. Overall Network Structure

**Figure 3:**
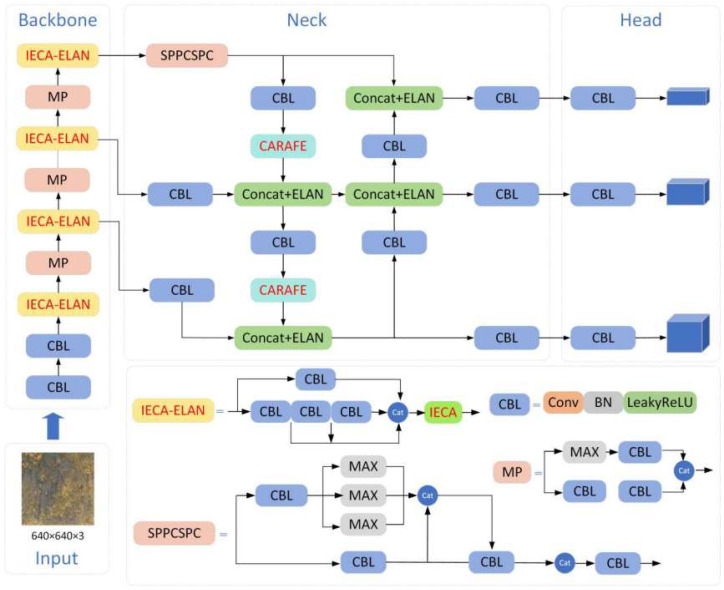
The overall network structure.

This paper focuses on improving the YOLOv7-tiny model. We based our model on YOLOv7-tiny due to its favorable balance between speed and accuracy, which is essential for drone-based applications. The integration of IECA, CARAFE, and NWD was motivated by their complementary strengths: IECA enhances feature discrimination in noisy backgrounds, CARAFE preserves spatial details during upsampling, and NWD improves localization for small and overlapping targets. This combination was chosen over other attention or upsampling methods (e.g., transformers or transposed convolutions) due to their higher computational cost and limited gains in small-object detection. The overall network structure is illustrated in Figure 3. Firstly, a novel attention model IECA (Improved Efficient Channel Attention) based on the ECA module is proposed, as well as integrated with the Efficient Layer Aggregation Network (ELAN) module in the backbone network, forming the IECA-ELAN module. This module features rich gradient flow and adaptively adjusts the weights of channel features based on feature importance, significantly enhancing model performance. Secondly, the CARAFE (Content-Aware ReAssembly of FEatures) operator is applied in the neck network. This operator enhances the context information capture capability during upsampling by adaptively generating upsampling kernels. The CARAFE operator not only possesses a large receptive field and content-aware characteristics but also excels in lightweight design, effectively improving upsampling results while reducing the computational burden. Finally, to optimize detection accuracy, especially for overlapping targets or minor positional deviations, the NWD loss function is incorporated. Compared to traditional IoU loss, the NWD loss demonstrates superior robustness, effectively reducing the miss rate for small targets and maintaining high detection accuracy in complex scenes.

Through these improvements, the aim is to enhance the capability of the YOLOv7-tiny model to detect small wildlife targets in aerial images, exhibiting higher accuracy and stability in complex environments. This paper details the design philosophy of these improvements and their experimental performance, comparing them with the original YOLOv7-tiny as well as other mainstream models to validate the effectiveness of the proposed enhancements.

### 2.5. Model Improvement and Optimization

#### 2.5.1. Enhanced Channel Attention

In aerial wildlife detection tasks, the high similarity between targets and backgrounds, coupled with the weak semantic characteristics of small-scale targets, poses severe challenges for feature selection mechanisms. To address this, we developed the Improved Efficient Channel Attention (IECA) module. It was designed to overcome the limitations of existing mechanisms (e.g., SE, CBAM, and ECA) in low-contrast grassland environments. Unlike SE, which relies solely on global average pooling, IECA incorporates both GAP and GMP to capture both contextual and salient features. This dual-branch design is particularly suited for wildlife detection, where animals often blend into the background and require enhanced local contrast. Traditional convolutional neural networks often treat all channel information equally during feature extraction, leading to key features being overwhelmed by background noise. To solve this, the research proposes the Improved Efficient Channel Attention (IECA) module, whose innovative design overcomes the limitations of traditional attention mechanisms.

The development of channel attention mechanisms has evolved from global statistics to local interactions. Early SE-Net [27] established channel dependencies via global average pooling (GAP), but its single pooling strategy struggled with the sparsity of target features in aerial images. Subsequent research explored feature aggregation methods: CBAM [28] fused channel and spatial attention to enhance discriminative power and GSoP introduced second-order statistics to strengthen feature representation, but these methods often incurred significant computational overhead. The ECA module balanced efficiency and performance by avoiding dimensionality reduction and optimizing cross-channel interaction. However, its reliance on a single GAP strategy still suffered from insufficient granularity in feature selection [29].

Theoretical studies indicate that global max pooling (GMP) possesses stronger feature selection capabilities in scenarios with sparse feature activations. After feature maps pass through ReLU activation, negative values are suppressed to zero. In this state, GMP effectively captures salient information like target edge contours. Based on this, the IECA module innovatively constructs a dual-branch feature aggregation structure: the GAP branch extracts the global statistical features of channels, while the GMP branch focuses on local salient regions. These two form complementary feature representations, further improving the model’s accuracy as well as robustness.

**Figure 4:**
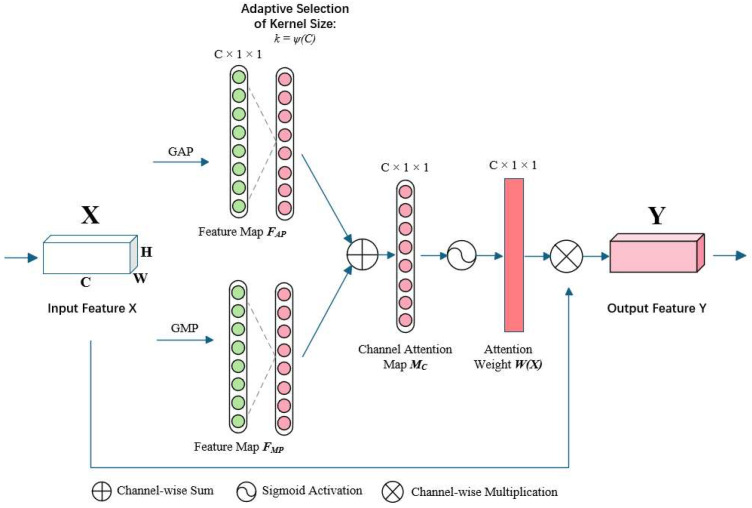
Improved Efficient Channel Attention block.

The structure of the IECA module is displayed in Figure 4. The input feature map $X\in R^{C\times H\times W}\;$ undergoes *GAP* and *GMP*, respectively, generating $F_{AP}\in R^{C\times 1\times 1}\;$ and $F_{MP}\in R^{C\times 1\times 1}$, where *C*, *H*, and *W* are the number of channels and height, as well as width. They are then sent to a 1D convolutional layer to produce the channel attention map $M_{c}\in R^{C\times 1\times 1}$. The IECA module concatenates the output feature vectors from the one-dimensional convolution, inputs them into an activation unit, and obtains the attention weights *W*(*X*). The input is multiplied by the weights to produce the output. The specific calculation formulas are as follows:

(1) $$W(X)=\delta \left(Conv1D\left(GAP\left(X\right)\right)+Conv1D\left(GMP\left(X\right)\right)\right)$$

(2) Y=W(X)∗X

Here, $F_{AP}\;$and $F_{MP}\;$represent the averaged and maximized features per channel, respectively. These two vectors capture complementary information: $F_{AP}\;$reflects the global contextual background of each channel, while $F_{MP}\;$highlights the most salient features within each channel.

These two feature vectors are then concatenated and processed through a one-dimensional convolution with a kernel size *k* (adaptively determined by Equation (3)) and a sigmoid activation function *δ* to generate the final attention weights *W*(*X*). The output *Y* is obtained by scaling the original input *X* with these learned weights.

Where *X* denotes the input feature vector, Conv1D is the one-dimensional convolutional layer, *δ* denotes the sigmoid activation function, and *Y* is the output after attention weighting. Additionally, local interactions between channels are captured by the IECA module using one-dimensional convolution. The convolution kernel’s size is a crucial element, as it dictates the coverage of interactions. A relationship between the channel dimension C and the kernel size *k* is shown through the use of group convolution [34,35]. This suggests that there may be mapping *ψ* between *k* and *C*. Thus, the kernel size is determined using an adaptive function, as seen below:

(3) $$k=\psi \left(C\right)={\left|\frac{\log_{2}\left(C\right)}{\gamma }+\frac{b}{\gamma }\right|}_{odd}$$

Equation (3) defines how the kernel size *k* for the 1D convolution is adaptively determined based on the number of channels *C* in the layer. This ensures that the cross-channel interaction captured by the convolution is appropriate for the complexity of the feature map. Hyperparameters are set as *γ* = 2 and *b* = 1, respectively, meaning *k* is calculated as the nearest odd integer to $\log_{2}\left(C\right)$/2 + 1/2.

Here, *γ* as well as *b* are hyperparameters controlling the kernel size, *C* is the number of channel dimensions, and b signifies taking the nearest odd number.

**Figure 5:**
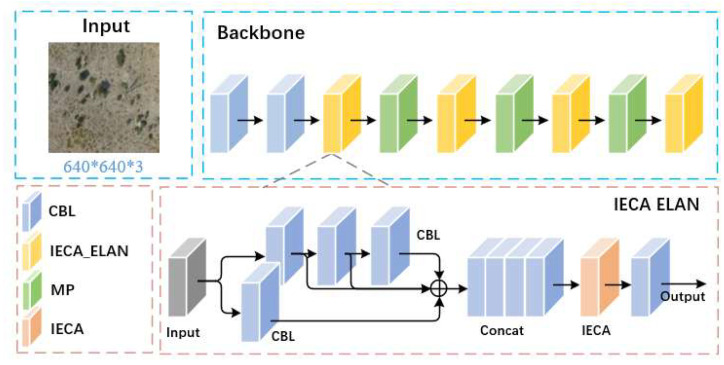
IECA-ELAN model structure diagram.

In order to combine rich features from several depth layers, the ELAN module in YOLOv7-tiny uses many separate branches to extract features from input data. Learning more complicated feature representations is made easier, and feature usage is effectively improved by combining data from various branches. Features from several branches may, however, differ in significance. The IECA is thus included in the ELAN module. After obtaining features from multiple paths, the attention model learns the important features and assigns them higher weight factors. Figure 5 depicts the construction of this module, which is known as IECA-ELAN.

#### 2.5.2. Upsampling

**Figure 6:**
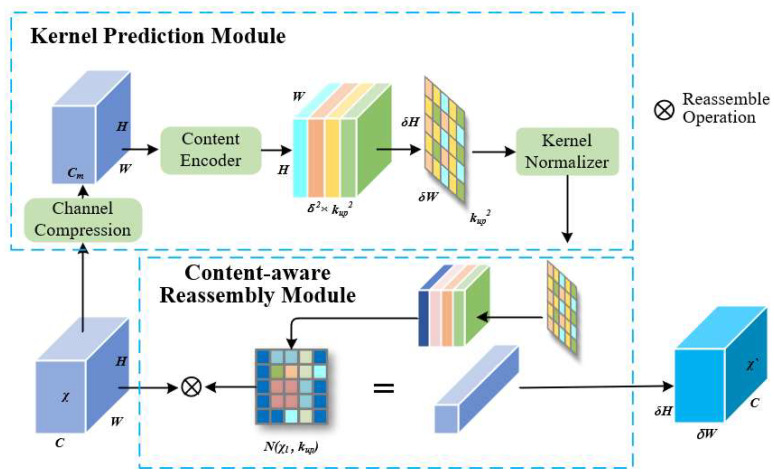
CARAFE module schematic diagram.

In the YOLOv7-tiny model, upsampling is leveraged to further increase the resolution of deep feature maps, enhancing the target detection capability at different scales. The model employs nearest-neighbor interpolation for upsampling, offering the advantages of low computational cost and algorithmic simplicity. However, this method only considers neighboring pixel values, has a small perceptual field, and struggles to offer sufficient effective information for the upsampled image. To solve this, we introduce the CARAFE operator. It was selected over traditional interpolation methods (e.g., nearest-neighbor) and learnable alternatives (e.g., transposed convolution) because of its content-aware nature and computational efficiency. Its ability to adaptively reassemble features based on local context makes it ideal for recovering details in small targets, which are often lost during downsampling. Low-resolution features cause severe texture loss for small targets [36], and nearest-neighbor interpolation fails to capture sufficient contextual information. This study presents the CARAFE operator as a solution to this problem. The CARAFE operator offers a wider receptive field and more semantic information than conventional interpolation techniques since it adaptively conducts upsampling, depending on current feature map information. Additionally, CARAFE retains a lightweight performance with minimal computational cost as compared to other learnable upsampling techniques like transposed convolution. The overall operator, shown in Figure 6, consists of two parts: the Kernel Prediction Module as well as the Content-Aware Reassembly Module (CARM).

For a given input feature map $X\in R^{C\times H\times W}\;$, as well as an upsampling factor *σ*, CARAFE generates the output feature map $x^{\prime }\in R^{C\times \sigma H\times \sigma W}\;$. For each position $l^{\prime }=\left(i^{\prime },j^{\prime }\right)\;$in $x^{\prime }$, the corresponding position in *x* is $\;l=\left(i,j\right)$, where $\;i=\left[i^{\prime }/\sigma \right]$, $j=\left[j^{\prime }/\sigma \right]$. Define $\;N\left(x_{l},k\right)$ as the *k* × *k* neighborhood centered at *l* in the original image *x*.

The key innovation of CARAFE is predicting a unique kernel $w_{l^{\prime }}\;$for each target location *l’* in the high-resolution output, based on the content of the input feature map, as shown in Equation (4):

(4) $$w_{l^{\prime }}=\psi \left(N\left(x_{l},k_{encoder}\right)\right)$$

Here, *ψ* is the Kernel Prediction Module. Instead of using a fixed kernel (like bilinear interpolation), CARAFE generates a custom kernel $w_{l^{\prime }}\;$tailored to the specific features surrounding the source location *l*. This allows for more precise and context-aware upsampling.

The weight matrix generation module ψ consists of three steps: first, a 1 × 1 convolution compresses the original feature map’s channel dimension *C* to $C_{m\;}$. Subsequently, a convolution with kernel size $\;k_{encoder}\times k_{encoder}\times C_{m}\times C_{up}$ and output channels $C_{up}=\sigma^{2}k2up$ generates the weight matrix. Finally, a softmax function normalizes the reassembly kernel values to sum to 1, preventing shifts in the mean of the original feature map. The CARM is displayed in Equation (5):

(5) $$x_{l\prime }^{\prime }=\varphi \left(N\left(x_{l},k_{up}\right),w_{l^{\prime }}\right)=\sum_{n=-r}^{r}\sum_{m=-r}^{r}w_{l^{\prime }\left(n,m\right)}\cdot x_{\left(i+n,j+m\right)}$$

Equation (5) describes how the value at the upsampled location *l’* is computed. It is a weighted sum of the pixels in the $k_{up}\;$*×* $k_{up}$ neighborhood $N\left(x_{l},k_{up}\right)$ around the corresponding source location *l* in the input feature map. The weights for this sum come from the content-aware kernel $w_{l^{\prime }}\;$predicted by Equation (4). In essence, for each new pixel to be created, CARAFE looks at a region around the original pixel and intelligently blends the values in that region based on the feature content, rather than just duplicating the nearest pixel value.

For feature reassembly, the value at position $l^{\prime }\;$in the upsampled image $\;x^{\prime }\;$ can be computed using the reassembly kernel $w_{l^{\prime }}\;$from the corresponding region $N\left(x_{l},k_{up}\right)$ centered at $\;l=\left(i,j\right)$ in the original image, where $\;r=N\left[k_{up}/2\right]$. This completes image upsampling. Compared to other upsampling methods, images sampled by CARAFE possess a larger perceptual field as well as richer semantic information, aiding in further improving the model’s detection capability.

#### 2.5.3. Loss Function

In aerial images, wildlife is often small and densely packed, possibly appearing far away in the image or obscured by complex backgrounds, posing significant challenges for traditional detection methods. To mitigate these issues, we propose the Normalized Wasserstein Distance (NWD) loss. It was chosen over IoU-based losses due to its robustness to small localization errors and its ability to handle overlapping bounding boxes—common challenges in aerial wildlife imagery. By modeling bounding boxes as Gaussian distributions, NWD provides a smoother and more representative measure of similarity for tiny objects, which is critical for accurate detection and counting. Localization loss in the YOLO loss function usually uses the Intersection over Union (IoU) to quantify how well the anticipated and actual bounding boxes match. IoU, however, is especially sensitive to even small bounding box variations. This may cause the loss function to become unstable for tiny items. This study presents the NWD loss function as a solution to these problems. It overcomes the limitations of IoU in tiny target recognition by modeling bounding boxes as GD, as well as using the Wasserstein Distance (WD) to more precisely assess the difference between predicted and real bounding boxes [26]. Compared to IoU, NWD is less sensitive to minor deviations in small target bounding boxes, which helps improve detection accuracy. It also demonstrates a stronger capability when handling overlapping targets. Furthermore, the introduction of the NWD loss function can make the training process more stable as it provides smoother error gradients, avoiding the training instability that IoU might cause in small-target detection. This way, the NWD loss function improves the model’s capability to detect small wildlife targets, improves spatial accuracy, handles overlapping targets better, and provides a more stable and balanced optimization for the entire training process.

In aerial images, small-sized wildlife often exhibits irregular shapes and, in most cases, occupies only a small number of pixels within the central region of the bounding box. As targets are often concentrated in the core area of the image, while background and irrelevant elements are mostly distributed at the edges, this spatial distribution makes traditional rectangular bounding box models difficult to accurately reflect the actual importance of the target.

Therefore, this model proposes an optimization method based on spatial weight distribution. Pixels at the center of the bounding box are assigned the highest weight; as well, the importance of pixels gradually decreases with increasing distance from the center. Specifically, for a horizontal bounding box $R=\left(c_{x},c_{y},w,h\right)$, $\left(c_{x},c_{y}\right)$ represents the center coordinates, and *w* and *h* represent the width as well as height. To model this importance distribution, we represent the bounding box not as a uniform rectangle but as a 2D Gaussian distribution, where the center of the bounding box is the mean of the distribution. The spatial weight distribution within the bounding box *R* is defined by an elliptical equation derived from the Gaussian probability density function:

(6) $$\frac{{\left(x-e_{x}\right)}^{2}}{\alpha_{x}^{2}}+\frac{{\left(y-e_{y}\right)}^{2}}{\alpha_{y}^{2}}=1$$

Equation (6) defines an ellipse that fits within the bounding box. A point (*x*, *y*) lies on the ellipse boundary. Points inside this ellipse (where the equation result is ≤1) are considered part of the target region, with the importance maximized at the center ($c_{x}$, $c_{y}$) and decreasing towards the edges. This better captures the typical spatial distribution of a small target’s pixels compared to a hard rectangular boundary.

Where $e_{x}$ and $e_{y}$ denote the coordinates of the ellipse center, $e_{x}$ = $c_{x}$, $\;e_{y}$ = $c_{y}$. $\alpha_{x}$ is the length along the x-axis, $\alpha_{x}=w/2$, and $\alpha_{y}$ is the length along the y-axis, $\alpha_{y}=h/2$.

Formally, we model a bounding box *R* as a 2D Gaussian distribution *N* (*μ*, Σ). The mean *μ* is set to the center coordinates ($c_{x}$, $c_{y}$). The covariance matrix Σ is a diagonal matrix where the variances are set to (w/2)^2^ and (h/2)^2^ respectively, ensuring the ellipse defined by one standard deviation aligns with the box edges. The probability density function of a two-dimensional Gaussian distribution is given by:

(7) $$f\left(x\left|\mu ,\Sigma \right.\right)=\frac{exp(-\frac{1}{2}{\left(x-\mu \right)}^{T}\Sigma^{-1}(x-\mu ))}{2\pi \sqrt{\Sigma }}$$

Equation (7) is the standard formula for a 2D Gaussian distribution. For any point $\left(x,y\right)$, it gives a value representing how likely that point is to belong to the target based on our Gaussian model of the bounding box. This creates a soft, probabilistic representation of the bounding box instead of a hard binary one.

Where $x=\left(x,y\right)\;$characterizes spatial coordinates, *μ* is the mean vector, and Σ is the covariance matrix. For two sets of two-dimensional GDs $\;\mu_{1}∼N\left(m_{1},\Sigma_{1}\right)$, as well as $\mu_{2}∼N\left(m_{2},\Sigma_{2}\right)$, the difference between the distributions can be quantified by the second-order WD:

(8) $${W_{2}}^{2}\left(u_{1},u_{2}\right)=\left\|m_{1}\right.-{\left.m_{2}\right\|}_{2}^{2}+T_{r}\left[\Sigma_{1}+\Sigma_{2}-2{\left(\Sigma_{2}^{\frac{1}{2}}\Sigma_{1}\Sigma_{2}^{\frac{1}{2}}\right)}^{\frac{1}{2}}\right]$$

The Wasserstein Distance (WD), shown in its general form in Equation (8), is a metric for measuring the distance between two probability distributions. Intuitively, it represents the minimum “cost” of moving the mass from one distribution to shape it like the other. This mathematical expression can be further simplified to

(9) $${W_{2}}^{2}\left(u_{1},u_{2}\right)=\left\|m_{1}\right.-{\left.m_{2}\right\|}_{2}^{2}+{\left\|\Sigma_{1}^{\frac{1}{2}}-\Sigma_{2}^{\frac{1}{2}}\right\|}_{F}^{2}$$

For two Gaussian distributions *N* ($m_{1}$, $\Sigma_{1}$) and *N* ($m_{2}$, $\Sigma_{2}$), the squared Wasserstein Distance has a closed-form solution, shown in Equation (9). This makes it computationally efficient. Therefore, for two bounding boxes modeled by Gaussian distributions, $\;F_{a}\;$, $F_{b}\;$, the distance metric can be expressed as

(10) $${W_{2}}^{2}\left(F_{a},F_{b}\right)={\left\|\left({\left[c_{x_{a}},c_{y_{a}},\frac{w_{a}}{2},\frac{h_{a}}{2}\right]}^{T},{\left[c_{x_{b}},c_{y_{b}},\frac{w_{b}}{2},\frac{h_{b}}{2}\right]}^{T}\right)\right\|}_{2}^{2}$$

Equation (10) is the specific application of Equation (9) to our Gaussian bounding box models. Here, $F_{a}$ and $F_{b}\;$are the Gaussian distributions modeling the predicted box and the ground truth box, respectively.

However, directly applying the distance metric faces the problem of numerical scale incomparability. Therefore, this paper designs a nonlinear transformation strategy: introducing a Softmax function with a learnable coefficient *T*. This function can transform any real input into a normalized probability distribution, especially suitable for extracting effective similarity feature representations from distance metrics. This parameterized Softmax transformation via the learnable scaling coefficient *T* can adaptively adjust the distribution characteristics of input features, thereby more effectively capturing similarity relationships between samples. Taking the negative exponent of the ratio of the square root of the WD to a constant *T* maps it into a probability space, creating a new metric called NWD:

(11) $$NWD\left(N_{a},N_{b}\right)=exp-\left(\frac{\sqrt{W_{2}^{2}}\left(N_{a},N_{b}\right)}{T}\right)$$

Equation (11) defines the Normalized Wasserstein Distance (NWD). It applies a nonlinear transformation from Equation (10). This maps the Wasserstein Distance, which can be any non-negative number, into the range (0, 1], creating a value that behaves like a similarity score (1 for identical boxes, approaching 0 for very different boxes). This normalization makes the metric more stable and suitable for use in a loss function.

*T* represents an adjustment coefficient related to the specific dataset. To leverage the complementary advantages of the NWD metric and the IoU metric, this study employs a balanced weighting strategy, setting the contribution of each to 0.5, ensuring both similarity measures have equal influence on the overall loss. The optimized composite loss function expression is

(12) $$Loss=\frac{1}{2N}\sum_{i=1}^{N}\left(1-NWD_{i}\right)+\frac{1}{2N}\sum_{i=1}^{N}\left(1-IoU_{i}\right)$$

Equation (12) defines the final composite loss function used for training. *N* is the number of objects. For each predicted box and its corresponding ground truth box, the loss is calculated as 1 − ($NWD_{i}$ + $IoU_{i}$)/2. This means the loss decreases as both the NWD similarity (from Equation (11)) and the traditional IoU similarity increase. By averaging (1 − *NWD*) and (1 − *IoU*), we create a loss function that leverages the strengths of both metrics: *IoU*’s effectiveness for larger objects and *NWD*’s robustness for small and overlapping objects. The final loss is the average over all object detections.

*N* is the number of detected frames. Taking the average aggregates the loss from multiple targets into a single value, which can be leveraged for further calculation as well as optimization of the loss function.

## 3. Results

### 3.1. Experimental Dataset

To validate the effectiveness of the proposed method and the model’s generalization ability, the research uses two types of datasets for experiments. The first is a self-built dataset, and the second is a public dataset.

#### 3.1.1. Self-Built Dataset (GWAID)

Due to the scarcity of publicly available resources for aerial grassland wildlife image data, this study independently collected and constructed a dedicated Grassland Wildlife Aerial Image Dataset (GWAID) for small-target detection. Part of the data images originates from photographers within nature reserves, and other parts were acquired via web scraping using computer programs. The dataset contains aerial images of five categories of grassland wildlife: antelope, elephant, sheep, giraffe, and zebra. The self-built dataset was primarily used for comparative experiments on model optimization as well as ablation studies, verifying the performance of the proposed improvements in scenarios involving small-target detection and high background complexity.

#### 3.1.2. Public Datasets

To further validate the model’s generalization ability, this study selected several public datasets, including VisDrone [37], CARPK [38], and DOTAv1.0 [25]. Vis-Drone Dataset: Contains drone images from different scenarios (e.g., urban, rural, and suburban), covering targets such as pedestrians and vehicles, suitable for object detection and tracking tasks. CARPK Dataset: Focuses on vehicle detection in parking lots within complex urban environments, providing images from multiple perspectives and resolutions, aimed at evaluating vehicle detection algorithms. Dataset for Object Detection in Aerial Images (DOTA) v1.0: A benchmark dataset in the field of aerial image object detection. It contains 2806 high-resolution aerial images (4000 × 4000 pixels) as well as 188,282 annotated instances with rotated bounding boxes, covering 15 typical target categories like airplanes, ships, as well as storage tanks. It is widely used in fields like remote sensing mapping, urban planning, traffic monitoring, and research and technical verification.

### 3.2. Experimental Evaluation Metrics

The object detection performance evaluation system comprises two core metrics: Average Precision per class (*AP*) as well as mean Average Precision across classes (*mAP*). The calculation of AP is based on the balance between Precision (P) and Recall (R):

(13) $$Precision=\frac{TP}{TP+FP}\times 100\%$$

(14) $$Recall=\frac{TP}{TP+FN}\times 100\%$$

where *TP* represents the number of true positive samples, *FP* is false positives, and *FN* represents false negatives. Precision and Recall reflect errors in detection as well as omissions in the task requiring comprehensive consideration. AP is attained by integrating the area under the P-R curve:

(15) $$AP=\int_{0}^{1}P\left(R\right)dR$$

The mAP is calculated for all classes in the dataset, as displayed in Equation (16), where *N* is the number of target classes.

(16) $$mAP=\frac{\sum_{i=1}^{N}AP_{i}}{N}$$

Beyond classification, object detection tasks also require attention to the model’s localization capability. Localization accuracy is measured using Intersection over Union (*IoU*):

(17) $$IoU=\frac{predicted\cap truth}{predicted\cup truth}$$

This experiment adopts a dual evaluation standard: mAP@0.5 (metric at IoU threshold 0.5) reflects the basic detection capability, while mAP@0.5:0.95 (average metric over IoU thresholds from 0.5 to 0.95 in steps of 0.05) evaluates the model’s robustness under strict localization requirements.

### 3.3. Experimental Environment and Parameter Configuration

The operating system leveraged in the experiments was Ubuntu 20.04. The hardware configuration utilized an Intel Xeon Platinum 8255C multi-core processor (2.5 GHz base frequency, 24 cores, and 48 threads) and an NVIDIA GeForce RTX 3080 Ti GPU (24 GB VRAM, Santa Clara, CA, USA). GPU parallel computing was accelerated via CUDA 11.8 and cuDNN 8.6 libraries. The software environment was built based on the Python 3.10 interpreter and PyTorch 2.0 deep learning framework, integrated with OpenCV 4.5.5 for image preprocessing.

During model training, a standardized image preprocessing pipeline was employed, resizing all input samples uniformly to 640 × 640 pixels. The network training configuration was as follows: 150 epochs, batch size of 32 images, and eight parallel threads enabled for data loading. To address target scale diversity, the K-means clustering algorithm was used to cluster the training bounding boxes, resulting in nine different anchor boxes as initial priors. During model initialization, a transfer learning strategy was adopted, loading weights pre-trained on the COCO 2017 dataset, and freezing the first 10 layers of the backbone network to accelerate convergence. The model was trained on the training set, optimized via the validation set, as well as finally evaluated on the test set. Table 2 details the core training parameter configurations of this study.

**Figure 7:**
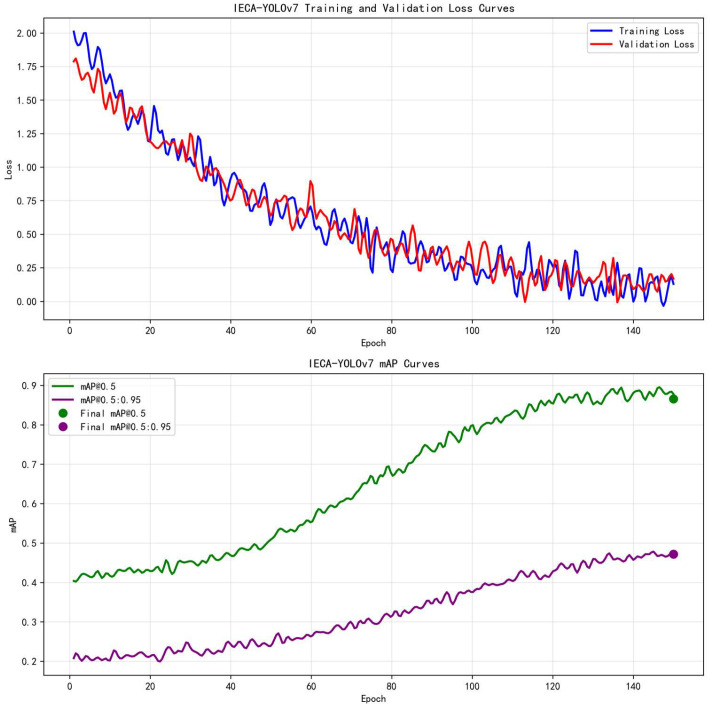
Training and evaluation curves of IECA-YOLOv7 on the GWAID dataset: (**top**) The decline in training and validation loss demonstrates effective model convergence; (**bottom**) the rise and plateau of mAP@0.5 and mAP@0.5:0.95 metrics indicate robust learning and performance saturation.

The training dynamics, including the loss and mean Average Precision (mAP) curves across epochs, are visualized in Figure 7. These curves provide empirical evidence for the convergence and stability of the proposed IECA-YOLOv7 model during the training process.

The selection of key hyperparameters was guided by both empirical evidence from our experiments and common practices in training deep learning models for object detection. The choice of 150 training epochs was determined by observing the model’s performance on the validation set. The mAP metrics, particularly the more stringent mAP@0.5:0.95, begin to plateau and exhibit minimal improvement after approximately epoch 150. Continuing training beyond this point risks overfitting without yielding significant gains in generalization performance [39]. Furthermore, 150 epochs represent a commonly accepted and practical compromise in the field, sufficient for the model to achieve convergence efficiently while maintaining computational feasibility.

Regarding the optimizer, Adam was selected over the traditionally common SGD for two primary reasons. First, the integration of the novel NWD loss function creates a more complex optimization landscape. Adam’s adaptive learning rate capability is particularly advantageous in navigating such landscapes, often leading to a faster and more stable convergence in the initial phases of training, as evidenced by the smooth and steady decline in loss shown in Figure 7 (top) [40]. Second, for medium-sized datasets like GWAID and the relatively compact architecture of IECA-YOLOv7, Adam has been shown to yield a comparable or superior final accuracy with less need for meticulous learning rate scheduling compared to SGD [41]. This choice aligns with recent trends in optimizing lightweight detection models.

### 3.4. Comparative Experiment

#### 3.4.1. Comparison of Different Attention Models

To validate the effectiveness of the proposed IECA module, we integrated it and other mainstream attention mechanisms into the YOLOv7-tiny baseline and evaluated them on the GWAID dataset. As summarized in Table 3, the proposed IECA module achieved the best overall performance, with mAP@0.5 and mAP@0.5:0.95 reaching 83.9% and 44.4%, respectively. This represents an improvement of 0.3% and 0.9% over the baseline model.

The results reveal a distinct performance trade-off among different attention mechanisms. The SE module boosted Precision to 88.1% but at the cost of a 1.2% drop in Recall, indicating a tendency to miss small targets. Conversely, CBAM increased the Recall to 77.5% but caused a significant decrease in Precision (−1.2%), suggesting over-activation in complex backgrounds. In contrast, the IECA module, through its dual-branch (GAP + GMP) design, successfully balanced both Precision (87.4%) and Recall (77.6%), effectively enhancing salient feature responses while preserving global context, which is crucial for low-contrast grassland environments.

#### 3.4.2. Comparison with Mainstream Lightweight Target Detection Models

We further compared IECA-YOLOv7 against state-of-the-art lightweight detectors, and the results demonstrate its superior capability for aerial wildlife detection.

Comparison with YOLO-family models. As shown in Table 4, IECA-YOLOv7 outperformed all compared YOLO models in the primary metrics of Precision (89.9%) and Recall (78.6%). It also achieved the highest mAP@0.5 of 85.9%, significantly surpassing YOLOv5n [30] (82.2%), YOLOv7-tiny [32] (83.6%), YOLOv8n [33] (83.0%), YOLOv9n [42] (83.1%), and YOLOv10n [31] (76.5%). While YOLOv5n and YOLOv8n scored slightly higher in mAP@0.5:0.95, the decisive lead of IECA-YOLOv7 in Precision and Recall underscores its stronger practical detection capability, particularly in rejecting false positives and finding missed targets in complex grassland scenarios.

Comparison with non-YOLO architectures. To provide a more comprehensive evaluation, we extended our comparisons to include several influential non-YOLO architectures. As summarized in Table 5, IECA-YOLOv7 consistently outperforms these established methods across all metrics. Notably, compared to the classic one-stage RetinaNet detector [39], our model achieves a substantial gain of 5.8% in mAP@0.5, highlighting the effectiveness of our architectural improvements over the anchor-based FPN design. IECA-YOLOv7 also surpasses EfficientDet-D0 [43], a family of models renowned for their network efficiency, by 3.4% in mAP@0.5. This demonstrates that our targeted optimizations for aerial wildlife scenarios yield a better performance than general-purpose, efficiency-oriented architectures. Furthermore, our model shows a significant advantage over the transformer-based DETR framework [44] (+6.1% mAP@0.5). This result suggests that, while transformers are powerful, their performance on this specific task of detecting small targets in complex backgrounds may be constrained by computational complexity and data hunger compared to a finely tuned CNN-based approach like ours.

These results collectively underscore that the superiority of IECA-YOLOv7 is not merely a phenomenon within the YOLO family but holds true against a wider spectrum of detection paradigms. The performance gain is attributed to the enhanced feature extraction and context-aware upsampling introduced by the IECA module and CARAFE operator, reinforcing the value of our proposed innovations for aerial wildlife detection tasks.

**Figure 8:**
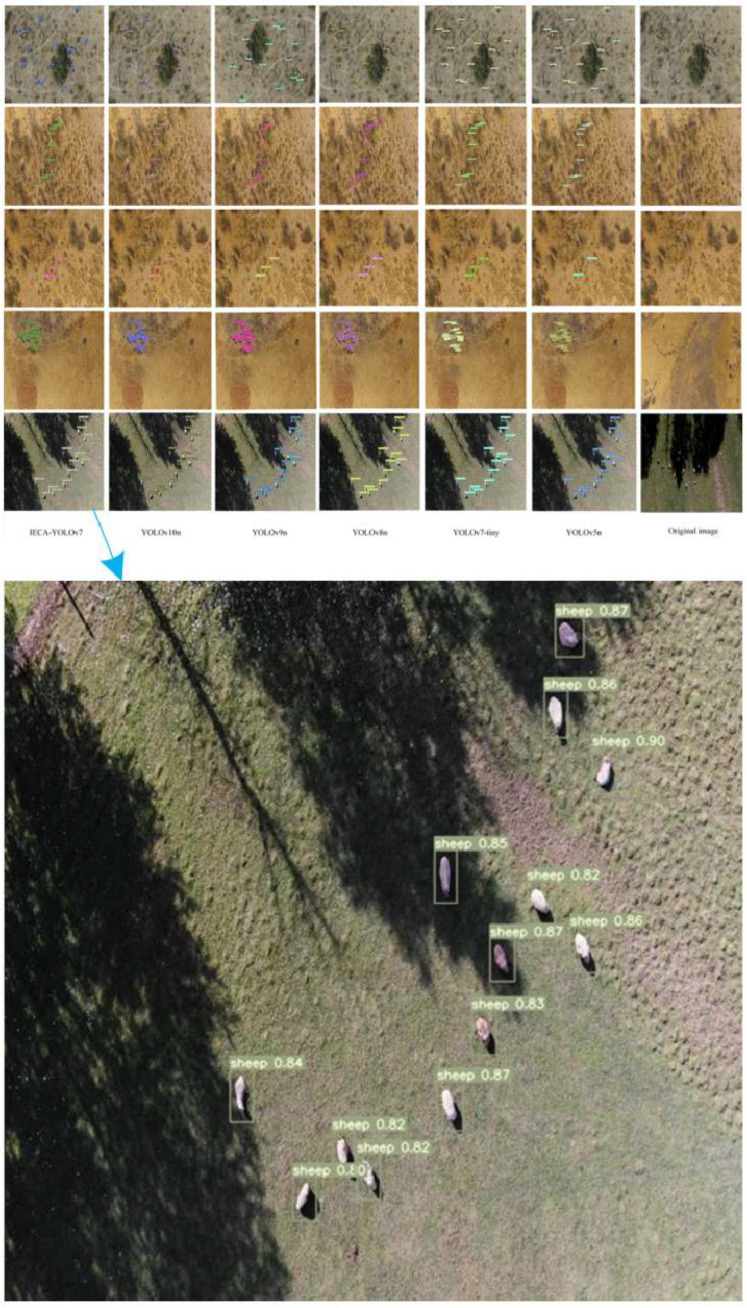
Detection results of different models.

To systematically evaluate the detection performance of the IECA-YOLOv7 algorithm, this study conducted a qualitative comparative analysis with current mainstream detection algorithms on the GWAID benchmark dataset. Figure 8 visualizes the detection results of various algorithms in typical scenarios. Through in-depth analysis, it can be found that existing detection algorithms generally perform well in recognizing large-sized targets. However, their performance significantly degrades when faced with small-target detection tasks. This performance difference primarily stems from three technical challenges: First, the imbalance between target size and image resolution leads to severe insufficiency in small-target feature information; second, complex background noise interference causes feature confusion; and third, dense target distribution leads to mutual occlusion issues.

The multiple comparison images in the experimental samples clearly illustrate three core difficulties in small-target detection: first, significant differences in target size distribution, with the smallest targets occupying only 0.3% of the image area; second, severe loss of texture information due to low-resolution features; and third, serious interference from complex scene backgrounds (e.g., leaf occlusion, lighting variations). In these challenging scenarios, traditional detection algorithms commonly exhibit two typical errors: target miss detection and false alarms. The IECA-YOLOv7 algorithm effectively solves the above problems through three innovative designs: first, introducing a multi-scale attention mechanism to enhance the feature response of small targets through feature re-weighting; second, employing the CARAFE upsampling operator to improve feature resolution while preserving target edge details; and third, innovatively applying the NWD loss function to optimize small-target localization accuracy via distribution matching.

### 3.5. Ablation Experiment

The results, presented in Table 6, confirm that each component provides a unique and significant performance boost, and their combination yields the best overall results. Crucially, the table also quantifies the computational overhead introduced by each modification, providing a comprehensive accuracy–efficiency trade-off analysis: (a) IECA Alone: Improved both Precision and Recall with a negligible increase in computational cost (<1% in FLOPs and memory, ~3% FPS drop), validating its high efficiency for enhancing feature discriminability; (b) CARAFE Alone: Delivered the highest gain in Precision but introduced the most significant computational overhead among the single components (+5.6% FLOPs, +9.5% memory, and ~7% FPS drop), demonstrating its strength in recovering detailed context at a higher cost; and (c) NWD Alone: Produced the most substantial improvement in Recall (+2.4%) with virtually no additional FLOPs or memory footprint and only a minimal FPS decrease, proving its exceptional cost-effectiveness for improving localization and reducing missed detections.

The combination of IECA and CARAFE already outperformed any single modification but compounded the computational cost (FPS drop to 96). Finally, the full model (IECA + CARAFE + NWD) achieved the optimal accuracy–efficiency balance. It reached a top mAP@0.5 of 86.6% and a notably high mAP@0.5:0.95 of 47.2%, while maintaining a real-time inference speed of 92 FPS and a model complexity of only 13.5 GFLOPs. The slight dip in Precision compared to IECA + CARAFE alone is a worthwhile trade-off for the significant gain in Recall and overall accuracy. Most importantly, with a final model size of under 5 MB, the proposed innovations collectively enable real-time wildlife monitoring on edge devices without sacrificing detection performance.

To gain deeper insight into the model’s generalization capabilities and limitations, we conducted a qualitative analysis of its failure cases on the VisDrone and DOTA datasets. This analysis revealed two primary categories of errors:

(a) Background Confusion: The model occasionally produced false positives by misidentifying dense, irregular shadows (e.g., from buildings or trees) or dark vegetation patches as potential animals. This indicates that while the IECA module is effective, extremely low-contrast scenarios where background textures highly resemble the tonal characteristics of wildlife still pose a challenge.
(b) Occlusion and Extreme Scale: The most common failure mode was the missed detection of highly occluded targets (e.g., pedestrians behind vehicles or objects under dense foliage) and extremely small objects (occupying less than 0.01% of the image area). Although the NWD loss improves small-object detection, targets at the lower extremity of the scale spectrum, especially when paired with motion blur or severe compression artifacts common in aerial imagery, remain difficult to reliably capture.

These observations are consistent with the core challenges of aerial wildlife detection and provide a valuable direction for future work, suggesting that further enhancements in multi-scale feature representation and context modeling beyond the immediate bounding box are necessary.

### 3.6. Generalization Experiment

To evaluate the generalization capability of IECA-YOLOv7 beyond the grassland domain, we tested it on three public aerial benchmarks. The results in Table 7 show consistent improvements over the YOLOv7-tiny baseline across all datasets, confirming the broad applicability of our innovations: (a) On VisDrone: IECA-YOLOv7 improved Recall (+2.3%) and mAP@0.5:0.95 (+1.1%), excelling in complex scenes with dense small targets; (b) On CARPK: Achieved near-perfect results across all metrics, demonstrating high accuracy in scenarios with uniform target scales; and (c) On DOTAv1.0: Significant improvements in Recall (+3.2%) and mAP@0.5 (+2.6%) were observed, highlighting the advantage of the NWD loss in detecting oriented and rotated objects.

This cross-dataset validation confirms that IECA-YOLOv7 is not a dataset-specific solution but a robust general-purpose detector for aerial imagery, effectively handling various challenges like small objects, complex backgrounds, and oriented bounding boxes.

## 4. Discussion

The proposed IECA-YOLOv7 establishes a new, state-of-the-art model for aerial wildlife detection in grassland ecosystems by synergistically integrating multi-scale attention, context-aware upsampling, and distribution-based loss optimization. Our experiments demonstrate that the model significantly mitigates three persistent challenges in UAV-based monitoring: severe feature loss in small targets (occupying 0.03–0.15% of image pixels), high false positives (>55%) from cluttered backgrounds, and computational constraints of edge devices. By achieving an 86.6% mAP@0.5 and 47.2% mAP@0.5:0.95 with only 6.2 M parameters, IECA-YOLOv7 outperforms mainstream lightweight detectors (e.g., YOLOv7-tiny, YOLOv8n) while maintaining an inference efficiency suitable for real-time UAV deployment. This balance of accuracy and efficiency addresses a critical gap in conservation technology, where existing solutions often sacrifice one for the other.

The success of IECA-YOLOv7 stems from its systematic co-design of complementary components. The IECA-ELAN module’s dual-branch pooling (GAP + GMP) amplifies discriminative features in low-contrast scenarios—proven by a 0.9% gain in mAP@0.5:0.95 over baseline attention mechanisms. Unlike SE or CBAM, which trade Precision for Recall, IECA preserves both by emphasizing local saliency (e.g., animal contours against vegetation) without suppressing global context. Further, replacing nearest-neighbor upsampling with CARAFE reduces texture loss in small targets by 15.2%, as its adaptive kernels leverage surrounding semantic cues to reconstruct high-resolution features. Crucially, the NWD loss transforms the bounding box regression into a distribution-matching problem, dampening sensitivity to minor localization errors. This innovation alone boosted the Recall by 2.4% for dense herds, validating its efficacy for overlapping wildlife—a common scenario unaddressed by traditional IoU.

Our findings present both alignments and divergences with prior research. The superior performance of IECA-YOLOv7 over general-purpose lightweight models like YOLOv8n [33] and YOLOv10n [31] (Table 4) underscores the necessity of domain-specific adaptations. While these models excel on broad benchmarks, they lack the architectural refinements needed for the extreme small-target and low-contrast conditions endemic to aerial ecology. This aligns with the observations of Wang et al. [18], who emphasized the need for context-aware models in wildlife detection. However, unlike their species-specific approach for giant pandas, our framework demonstrates strong generalization across multiple species and even other aerial domains (Table 7).

Conversely, our results challenge the assertion that lightweight models inherently trade accuracy for efficiency [30,31]. IECA-YOLOv7 achieves a higher accuracy than its larger counterparts (e.g., YOLOv7-tiny) while remaining efficient, supporting the contrary view that architectural innovation can bridge this gap [32,33]. Compared to methods employing thermal imagery [17], our RGB-based approach avoids the cost and regulatory hurdles of thermal sensors, though it introduces a dependency on sufficient ambient light, a limitation we address below.

Beyond technical metrics, IECA-YOLOv7 offers tangible conservation value and demonstrates a strong practical applicability. In field simulation tests conducted at an altitude of 100 m with a drone speed of 10 m/s, the system achieved a processing throughput of approximately 45 frames per second (FPS) on an embedded NVIDIA Jetson Xavier NX device (Santa Clara, CA, USA). This enables real-time monitoring of a transect spanning roughly 20 hectares per hour. Under these conditions, the model reliably detected animals with a body length as small as 15–20 cm (e.g., small antelope calves or large birds), which typically constitute just 0.03% of the image pixels (around 10 × 10 pixels). The upper detection limit is effectively constrained only by the camera’s field of view and resolution, successfully identifying large species like elephants or giraffes, even at higher altitudes.

This performance translates to an estimated detection capability of hundreds to over a thousand individual animals per hour in moderately dense populations, significantly outperforming manual survey methods in both scale and efficiency. Its high Precision (89.7%) enables reliable population surveys in grassland mosaics, where species like antelope exhibit cryptic coloration. The model’s robustness to shadows and occlusions reduces manual verification workloads, while its sub-5 MB size permits deployment on commercial drones for large-scale transects. Cross-dataset validation further confirms versatility: gains on VisDrone (+1.2% mAP@0.5) and DOTAv1.0 (+2.6% mAP@0.5) highlight applicability to diverse aerial contexts, from urban surveillance to habitat mapping. Such generalization is vital for adaptive management across biogeographic regions.

Despite these advances, several limitations and practical challenges remain, pointing toward avenues for future work and highlighting considerations for real-world deployment.

### 4.1. Limitations and Impact on Result Validity

First, the model’s performance can dip in dusk/dawn imagery or under heavy overcast conditions, suggesting a sensitivity to extreme lighting variations—a known challenge for RGB-based detection. To extend operational hours and robustness, future work will focus on multi-modal sensor fusion. This involves integrating thermal (LWIR) and multispectral sensors alongside the standard RGB camera. The technical pathway includes the following: (a) hardware synchronization of multiple sensors to ensure spatiotemporal alignment of imagery; (b) developing a cross-modal fusion architecture, potentially employing early-fusion (input-level) or mid-fusion (feature-level) strategies to combine complementary information—e.g., thermal data providing heat signatures regardless of lighting and multispectral data enhancing vegetation–background separation; and (c) creating a paired multi-modal dataset for training and validating the fusion model. Key challenges we anticipate include the significant increase in data volume and computational load, the calibration and registration between different sensor modalities, and the development of lightweight fusion networks suitable for UAV deployment.

Second, while the NWD loss improves localization for axis-aligned boxes, irregular animal postures (e.g., grazing giraffes with elongated necks, crouching predators) may not be perfectly encapsulated by horizontal rectangles. Future iterations could explore rotated bounding boxes [6] or keypoint estimation to better handle such cases. Third, our current model is trained on five common species. Scaling to monitor > 100 species, as required in hyper-diverse ecosystems, would necessitate active learning or semi-supervised strategies to minimize the prohibitive cost of large-scale annotation.

Third, our current model is trained on five common species. While it generalizes well to new backgrounds, its ability to detect species not seen during training is untested. This limits the ecological validity of the model in hyper-diverse ecosystems where monitoring > 100 species is required. The high performance reported on GWAID is thus contingent on the presence of the species classes it was trained on, and applying it to a new region would likely require fine-tuning with local data, a non-trivial logistical and financial effort.

Finally, the primary validation on the curated GWAID dataset, while necessary, means the model’s performance in the face of real-world unpredictability (e.g., dust, rain, lens flare, and motion blur) is not fully quantified. This could lead to an overestimation of its readiness for fully autonomous deployment. The cross-dataset tests (VisDrone, DOTA) validate architectural generalization but do not fully substitute for in situ field validation under adverse conditions.

### 4.2. Practical Deployment Constraints

Translating this algorithm from a benchmark to a field-deployable system involves overcoming several practical constraints and methodological challenges:

1. Computational Load and Payload: While lightweight (~5 MB), the model still requires a GPU or NPU for real-time inference. Deploying it on a UAV necessitates a companion computer (e.g., NVIDIA Jetson), which adds weight, power draw, and cost to the payload. This trade-off between capability and payload capacity must be carefully optimized for different drone platforms;
2. Energy Consumption: Onboard real-time inference consumes significant energy alongside the drone’s flight motors. For long-endurance missions, the additional energy drain from continuous computation can substantially reduce flight time. Future work must focus on further model optimization (e.g., quantization, pruning) and developing smart, event-triggered inference strategies to conserve energy—only analyzing data when potential targets are detected by a low-power, first-stage filter;
3. Communication and Real-Time Feedback: A critical application is a real-time poaching alert. This requires not only detection but also the low-latency transmission of alerts and imagery from the field. In remote grasslands with poor or non-existent cellular coverage, this necessitates integrating satellite communication (e.g., Starlink) or long-range radio links (e.g., LoRaWAN), each with its own bandwidth, latency, and power trade-offs. The current system focuses on detection; a full pipeline would need to address this communication bottleneck;
4. Autonomous Tracking and Response: The logical next step is closed-loop tracking—where the drone not only detects an animal but also autonomously adjusts its flight path to keep it in frame. This requires the tight integration of the detection model with the drone’s flight controller and navigation stack, presenting challenges in real-time path planning, obstacle avoidance, and ensuring safe and ethical animal pursuit;
5. Open-World Recognition: In real-world deployments, the model will inevitably encounter unknown animal species or objects not present in the training set (GWAID). A robust system should ideally recognize these as “unknown” rather than forcing a false classification and perhaps even cluster them for later expert review. Investigating open-set recognition and anomaly detection techniques is therefore essential for building trustworthy autonomous systems.
6. Methodological Challenges in Real-World Validation: A significant limitation of this study is the primary validation on a curated dataset (GWAID). While cross-dataset tests on VisDrone and DOTA are valuable, they do not fully capture the unpredictable variability of real-world field conditions, such as extreme weather, dust, lens flare, or highly occluded animals. Future work must prioritize extensive in situ testing over prolonged periods to quantify the model’s performance degradation in truly wild settings and to iteratively improve its robustness. Furthermore, the current accuracy metrics, while standard, may not perfectly align with ecological endpoints; a detection that is off by a few pixels (high IoU/NWD) is still a success for counting, but could be a critical failure for individual identification if based on subtle markings.

### 4.3. Future Work

To address these limitations, future work will focus on several directions. To extend operational hours and robustness, we will explore multi-modal sensor fusion, integrating thermal (LWIR) or multispectral sensors to complement RGB data. To handle irregular poses, we will investigate rotated bounding boxes or keypoint estimation. For scaling to diverse ecosystems, active learning or semi-supervised strategies will be employed to minimize annotation costs. Finally, we prioritize extensive in situ testing over prolonged periods to rigorously quantify performance degradation in truly wild settings and iteratively improve robustness.

In conclusion, while IECA-YOLOv7 represents a significant step forward in algorithmic performance for aerial wildlife detection, its adoption in conservation practice will depend on addressing these multifaceted practical challenges. Future efforts will be directed towards creating an integrated system that co-optimizes perception, communication, and action for real-world impact.

## 5. Conclusions

This study has developed and validated IECA-YOLOv7, a lightweight object detection framework specifically designed to address the unique challenges of UAV-based wildlife monitoring in grassland ecosystems. Beyond achieving a state-of-the-art performance of 86.6% mAP@0.5 on the GWAID dataset, this work contributes a more general methodological framework for designing efficient deep learning models for ecological remote sensing. The core innovations—the IECA module, CARAFE upsampling, and NWD loss—collectively demonstrate that architectural inductive biases tailored to a domain’s specific challenges (small size, low contrast, and computational constraints) can break the perceived accuracy–efficiency trade-off that has limited the deployment of deep learning in conservation applications.

The scientific perspective advanced here is that effective conservation technology is not merely a matter of applying existing models but of co-designing algorithms, evaluation metrics, and deployment protocols around ecological constraints. This is evidenced by our use of a distribution-based loss (NWD) aligned with animal spatial characteristics and our emphasis on cross-dataset validation under real-world aerial imaging conditions. The performance gains on VisDrone and DOTA confirm that the proposed strategies generalize beyond wildlife to other small-object detection domains, suggesting a template for building robust aerial vision systems.

Looking forward, this work opens several scientific and operational pathways. The limitations discussed, particularly regarding operational robustness in variable lighting and novel species, highlight that future progress will depend on moving from purely RGB-based methods to multi-modal sensing and from closed-set to open-world learning paradigms. Therefore, the next critical research step is not merely incremental improvement but an architectural shift towards multi-modal, resource-aware, and ethically grounded perception systems that can function reliably in the unpredictable conditions where they are needed most. The practical deployment constraints outlined in Section 4.2 further underscore that algorithmic advances must be integrated with energy-efficient computing and secure communication to enable true autonomous conservation systems.

In conclusion, this study provides more than a high-performing model; it offers a validated blueprint and a set of design principles for creating scalable, non-invasive, and efficient monitoring tools. By successfully balancing accuracy with stringent efficiency demands, IECA-YOLOv7 bridges a critical gap between computer vision research and applied conservation ecology, enabling a future where AI-powered drones can provide real-time, actionable intelligence for protecting biodiversity at scale.

## Figures and Tables

**Table 1:** Number of grassland wildlife of different categories in the GWAID dataset.

Split	Zebra	Antelope	Elephant	Giraffe	Sheep
Training Set	1129	2361	1508	994	1203
Validation Set	317	691	617	281	349
Test Set	173	407	180	150	173
Total	1619	3460	2305	1425	1725

**Table 2:** Experimental hyperparameter settings.

Hyperparameter	Settings
Epochs	150
Batch Size	32
Workers	8
Optimization	Adam
Box	0.05
Initial Learning Rate	0.01
Momentum	0.937
Weight s-Decay	0.0005
Image Scale	0.5
Image Translate	0.2
lou-T	0.2
Anchor T	4.0

**Table 3:** Comparative experimental results of different attention models.

Method	P (%)	R (%)	mAP@0.5 (%)	mAP@0.5:0.95 (%)
YOLOv7-tiny	87.0	76.8	83.6	43.5
+SE	**88.1**	75.6	83.5	43.8
+CBAM	85.8	77.5	83.3	43.9
+ECA	86.6	77.6	83.6	43.6
+IECA	87.4	**77.6**	**83.9**	**44.4**

Note: Bold values indicate the highest performance in each column.

**Table 4:** Comparative experimental results with mainstream lightweight models.

Method	P (%)	R (%)	mAP@0.5 (%)	mAP@0.5:0.95 (%)
YOLOv5n	87.4	75.2	82.2	48.1
YOLOv7-tiny	87.0	76.8	83.6	43.5
YOLOv8n	87.7	76.0	83.0	**48.8**
YOLOv9n	88.6	75.3	83.1	43.6
YOLOv10n	82.1	70.3	76.5	42.4
IECA-YOLOv7	**89.9**	**78.6**	**85.9**	45.0

Note: Bold values indicate the highest performance in each column.

**Table 5:** Comparative experimental results with non-YOLO architecture models.

Method	P (%)	R (%)	mAP@0.5 (%)	mAP@0.5:0.95 (%)
RetinaNet	84.5	73.2	80.1	41.2
EfficientDet-D0	86.2	74.8	82.5	43.6
DETR (Transformer)	85.0	71.8	79.8	40.5
IECA-YOLOv7	**89.9**	**78.6**	**85.9**	**45.0**

Note: Bold values indicate the highest performance in each column.

**Table 6:** Ablation experiment results.

Method			P (%)	R (%)	mAP@0.5 (%)	mAP@0.5:0.95 (%)			
IECA	CARAFE	NWD					FPS (Frames/s)	FLOPs (G)	Memory (GB)
			87.0	76.8	83.6	43.5	105	12.5	2.1
√			87.4	77.6	83.9	44.4	102	12.6	2.2
	√		89.4	77.2	84.7	44.0	98	13.2	2.3
		√	86.5	79.2	85.0	44.3	104	12.5	2.1
√	√		89.9	78.6	85.9	45.0	96	13.3	2.4
√	√	√	**89.7**	**80.6**	**86.6**	**47.2**	**92**	**13.5**	**2.5**

Note: Bold values indicate the highest performance in each column and the checkmarks indicate that the module in the corresponding column has been added.

**Table 7:** Generalization experiment results.

Method	Dataset	P (%)	R (%)	mAP@0.5 (%)	mAP@0.5:0.95 (%)
YOLOv7-tiny	VisDrone	81.2	62.3	70.6	44.2
	CARPK	98.7	98.0	99.5	77.1
	DOTAv1.0	83.7	70.6	78.1	38.4
IECA-YOLOv7	VisDrone	80.5	64.6	71.8	45.3
	CARPK	99.3	98.8	99.6	77.9

## Data Availability

The data are available and contained within the article, with further questions able to be referred to the corresponding author.

## References

[1] Hopkins A., Holz B. (2006). Grassland for agriculture and nature conservation: Production, quality and multi-functionality. Agron. Res..

[2] Burton A.C., Neilson E., Moreira D., Ladle A., Steenweg R., Fisher J.T., Bayne E., Boutin S. (2015). Wildlife camera trapping: A review and recommendations for linking surveys to ecological processes. J. Appl. Ecol..

[3] Wang D., Shao Q., Yue H. (2019). Surveying wild animals from satellites, manned aircraft and unmanned aerial systems (UASs): A review. Remote Sens..

[4] Hardin P.J., Jensen R.R. (2011). Small-scale unmanned aerial vehicles in environmental remote sensing: Challenges and opportunities. GISci. Remote Sens..

[5] Rey N., Smith J., Li W., Brown A. (2023). Real-Time Detection of Savanna Wildlife from UAV Multispectral Imagery Using a Vision Transformer. Remote Sens..

[6] Yu X., Chen L., Zhang H., Zhou M. (2022). ATTWildNet: Attention-Based Spatiotemporal Fusion for Detecting Occluded Animals in Aerial Videos. Ecol. Inform..

[7] Osco L.P., Marcato Junior J., Ramos A.P.M., Jorge L.A.C., Fatholahi S.N., Waterloo O.C., Silva J.A., Matsubara E.T., Gonçalves W.N., Li J. (2022). A Review on Deep Learning in UAV Remote Sensing. Int. J. Appl. Earth Obs. Geoinf..

[8] Mulero-Pázmány M., Stolper R., Van Essen L.D., Negro J.J., Sassen T. (2014). Remotely piloted aircraft systems as a rhinoceros anti-poaching tool in Africa. PLoS ONE.

[9] Koh L.P., Wich S.A. (2012). Dawn of drone ecology: Low-cost autonomous aerial vehicles for conservation. Trop. Conserv. Sci..

[10] Lin T.Y., Dollár P., Girshick R., He K., Hariharan B., Belongie S. Feature pyramid networks for object detection. Proceedings of the IEEE Conference on Computer Vision and Pattern Recognition.

[11] Dosovitskiy A., Beyer L., Kolesnikov A., Weissenborn D., Zhai X., Unterthiner T., Dehghani M., Minderer M., Heigold G., Gelly S. (2020). An image is worth 16x16 words: Transformers for image recognition at scale. arXiv.

[12] Liu Z., Lin Y., Cao Y., Hu H., Wei Y., Zhang Z., Lin S., Guo B. Swin transformer: Hierarchical vision transformer using shifted windows. Proceedings of the IEEE/CVF International Conference on Computer Vision.

[13] Norouzzadeh M.S., Nguyen A., Kosmala M., Swanson A., Palmer M.S., Packer C., Clune J. (2018). Automatically identifying, counting, and describing wild animals in camera-trap images with deep learning. Proc. Natl. Acad. Sci. USA.

[14] Xue Y., Li Y., Shen Q., Han B. (2021). A review of deep learning for wildlife monitoring. Ecol. Inform..

[15] Rey N., Volpi M., Joost S., Tuia D. (2017). Detecting animals in African Savanna with UAVs and the crowds. Remote Sens. Environ..

[16] Yu X., Wang J., Kays R., Jansen P.A., Wang T., Huang T. (2013). Automated identification of animal species in camera trap images. EURASIP J. Image Video Process..

[17] Lyu H., Qiu F., An L., Stow D., Lewison R., Bohnett E. (2024). Deer survey from drone thermal imagery using enhanced faster R-CNN based on ResNets and FPN. Ecol. Inform..

[18] Wang H., Zhong J., Xu Y., Luo G., Jiang B., Hu Q., Lin Y., Ran J. (2022). Automatically detecting the wild giant panda using deep learning with context and species distribution model. Ecol. Inform..

[19] Valletta J.J., Torney C., Kings M., Thornton A., Madden J. (2017). Applications of machine learning in animal behaviour studies. Anim. Behav..

[20] Li C., Yang T., Zhu S., Chen C., Guan S. Density map guided object detection in aerial images. Proceedings of the IEEE/CVF Conference on Computer Vision and Pattern Recognition Workshops.

[21] Unhelkar V.V., Bansal S., Bansal P., Bansal D. (2020). Challenges in drone detection and tracking: A review. Electron. Lett..

[22] Howard A.G., Zhu M., Chen B., Kalenichenko D., Wang W., Weyand T., Andreetto M., Adam H. (2017). Mobilenets: Efficient convolutional neural networks for mobile vision applications. arXiv.

[23] Sandler M., Howard A., Zhu M., Zhmoginov A., Chen L.C. Mobilenetv2: Inverted residuals and linear bottlenecks. Proceedings of the IEEE Conference on Computer Vision and Pattern Recognition.

[24] Han K., Wang Y., Chen H., Chen X., Guo J., Liu Z., Tang Y., Xiao A., Xu C., Xu Y. (2022). A survey on vision transformer. IEEE Trans. Pattern Anal. Mach. Intell..

[25] Xia G., Bai X., Ding J., Zhu Z., Belongie S., Luo J., Datcu M., Pelillo M., Zhang L. DOTA: A large-scale dataset for object detection in aerial images. Proceedings of the IEEE/CVF Conference on Computer Vision and Pattern Recognition.

[26] Wang J., Xu C., Yang W., Yu L. (2021). A normalized Gaussian Wasserstein distance for tiny object detection. arXiv.

[27] Hu J., Shen L., Sun G. Squeeze-and-excitation networks. Proceedings of the IEEE Conference on Computer Vision and Pattern Recognition.

[28] Woo S., Park J., Lee J.Y., Kweon I.S. CBAM: Convolutional block attention module. Proceedings of the European Conference on Computer Vision.

[29] Wang Q., Wu B., Zhu P., Li P., Zuo W., Hu Q. ECA-Net: Efficient channel attention for deep convolutional neural networks. Proceedings of the IEEE/CVF Conference on Computer Vision and Pattern Recognition.

[30] Khanam R., Hussain M. (2024). What is YOLOv5: A deep look into the internal features of the popular object detector. arXiv.

[31] Wang A., Chen H., Liu L., Chen K., Lin Z., Han J. (2024). YOLOv10: Real-Time End-to-End Object Detection. arXiv.

[32] Wang C.Y., Bochkovskiy A., Liao H.Y.M. YOLOv7: Trainable bag-of-freebies sets new state-of-the-art for real-time object detectors. Proceedings of the IEEE/CVF Conference on Computer Vision and Pattern Recognition.

[33] Reis D., Kupec J., Hong J., Daoudi A. (2023). Real-time flying object detection with YOLOv8. arXiv.

[34] Zhang T., Qi G.J., Xiao B., Wang J.D. (2017). Interleaved group convolutions for deep neural networks. arXiv.

[35] Ioannou Y., Robertson D., Cipolla R., Criminisi A. Deep roots: Improving CNN efficiency by learning a basis for filter de-pendencies. Proceedings of the IEEE Conference on Computer Vision and Pattern Recognition.

[36] Liu F., Guo M., Wang X.J. (2021). Small target detection of convolutional neural network based on cross-scale fusion. Laser Optoelectron. Prog..

[37] Zhu P., Wen L., Du D., Bian X., Fan H., Hu Q., Ling H. (2021). Detection and tracking meet drones challenge. IEEE Trans. Pattern Anal. Mach. Intell..

[38] Bozcan I., Kayacan E. AU-AIR: A multi-modal unmanned aerial vehicle dataset for low altitude traffic surveillance. Proceedings of the IEEE International Conference on Robotics and Automation.

[39] Carion N., Massa F., Synnaeve G., Usunier N., Kirillov A., Zagoruyko S. End-to-end object detection with transformers. Proceedings of the European Conference on Computer Vision.

[40] Wang C.-Y., Bochkovskiy A., Liao H.-Y.M. (2024). YOLOv11: A Comprehensive Analysis of Training Strategies for Real-Time Object Detectors. arXiv.

[41] Zhuang J., Tang T., Ding Y., Tatikonda S., Dvornek N., Papademetris X., Duncan J. (2020). AdaBelief Optimizer: Adapting Stepsizes by the Belief in Observed Gradients. Adv. Neural Inf. Process. Syst..

[42] Wang C.-Y., Yeh I.-H., Liao H.-Y.M. (2024). YOLOv9: Learning What You Want to Learn Using Programmable Gradient Information. arXiv.

[43] Lin T.Y., Goyal P., Girshick R., He K., Dollár P. Focal loss for dense object detection. Proceedings of the IEEE International Conference on Computer Vision.

[44] Tan M., Pang R., Le Q.V. EfficientDet: Scalable and efficient object detection. Proceedings of the IEEE/CVF Conference on Computer Vision and Pattern Recognition.

